# Strengthening of Masonry and Concrete Members with Textile-Reinforced Alkali-Activated Mortars: A Review on the Mechanical Performance

**DOI:** 10.3390/ma18071517

**Published:** 2025-03-28

**Authors:** Paraskevi D. Askouni, Panagiotis Kapsalis, Catherine G. Papanicolaou, Thanasis C. Triantafillou

**Affiliations:** 1 Service of Modern Monuments and Technical Works of Western Greece, Peloponnese and Southern Ionian Sea, Ministry of Culture, 26223 Patras, Greece; paskouni@culture.gr; 2 Department of Civil Engineering, University of Patras, 26504 Patras, Greece; pkapsalis@ac.upatras.gr (P.K.); kpapanic@upatras.gr (C.G.P.)

**Keywords:** alkali-activated mortar (AAM), concrete, masonry, strengthening, textile-reinforced mortar (TRM)

## Abstract

Textile-reinforced alkali-activated mortar (TRAAM) is a composite material that is characterized by a strain- or deflection-hardening response under tension or flexure, respectively, as well as by a good bond with concrete and masonry substrates. Owing to comparable or even superior mechanical performance compared to “conventional” cement- or lime-based textile-reinforced mortar (TRM) systems and its potentially eco-friendly energy and environmental performance, TRAAM has been incorporated to retrofitting schemes. The current article reviews the studies that investigate TRAAM as a strengthening overlay for masonry and concrete members. This article focuses on the mechanical performance of the strengthened members, which, where possible, is also compared with that of members strengthened with conventional TRM systems. It is concluded that TRAAM can enhance the flexural and shear capacity of masonry and concrete members, while it can also upgrade the compression strength and seismic response of concrete members. In addition, it is concluded that the effectiveness of TRAAM can be comparable with that of “conventional” TRM systems. The combination of TRAAM with thermal insulation boards has also been proposed for structural and energy upgrading of masonry walls. Furthermore, TRAAM can be a promising solution for increasing the fire resistance of strengthened masonry members. However, research on the long-term performance of TRAAM, including durability, creep, and shrinkage, is still limited. Finally, the lack of established standards for TRM retrofitting is more evident for TRAAM applications.

## 1. Introduction

### 1.1. Retrofitting with Textile-Reinforced Mortars (TRM)

As the global building stock ages, the need to retrofit existing structures becomes increasingly pressing. Retrofitting not only ensures the structural safety and longevity of buildings but also offers a sustainable and, usually, more economical alternative to complete demolition and reconstruction. Among various strengthening techniques, textile-reinforced mortars (TRM) have emerged, during the last 20 years or so, as an effective and versatile solution. These systems utilize fibrous non-metallic, thus non-corroding, reinforcement in the form of open-weave grids, with fiber rovings usually arranged in two orthogonal directions. The most commonly used matrices are either cement or lime based. TRM demonstrate superior mechanical properties, such as high tensile strength and ductility, while they also present sufficient bonding with typical substrates, such as concrete and masonry. Their effectiveness in enhancing the structural performance in terms of flexure, shear and confinement, as well as their ability to upgrade the seismic response regarding ductility and energy dissipation, has been validated through experimental and analytical studies and practical applications [1].

### 1.2. Retrofitting with Textile-Reinforced Alkali-Activated Mortars (TRAAM)

Conventional cementitious mortars, commonly used as matrix of TRM systems, significantly contribute to the overall carbon footprint buildup of construction materials due to the high energy demand and CO₂ emissions associated with the production of cement. This has led to a growing interest in developing more sustainable alternatives. Alkali-activated materials (AAM) have gained attention as a viable substitute of cement-based materials since a broad category of them comprise industrial waste or by-products (thus relating to significantly lowering greenhouse gas emissions during production). Specifically, AAM comprise aluminosilicate powders (precursors) such as fly ash and furnace slags, which are activated using alkaline solutions (activators) such as potassium- or sodium-based solutions. The resulting binders usually offer comparable or even superior mechanical properties to ordinary Portland cement [2].

The integration of AAM into TRM systems combines the benefit of a ‘green’ material technology (aligned with global efforts to mitigate climate change) with an innovative structural upgrading technique giving rise to a new family of composite materials, namely, textile-reinforced alkali-activated mortars (TRAAM), also known as TRG (textile-reinforced geopolymers). There are already several studies regarding the characterization of the physical/chemical properties, the mechanical behavior, the durability performance, as well as the energy and environmental performance of various TRAAM systems.

### 1.3. Tensile Performance of TRAAM

The uniaxial tensile behavior of TRAAM has been investigated by a limited number of studies. Candamano et al. (2020) [3] performed a physical, chemical and mechanical characterization of basalt fiber TRAAM made of a ternary blend (fly ash, blast furnace slag and forest biomass ash activated by a mix of potassium hydroxide and sodium trisilicate). The capillary absorption of the AAM was within the range of values corresponding to commercial lime- or cement-based matrices. However, the material’s free shrinkage could be further improved to be comparable with that of the commercial matrices characterized by higher dimensional stability. The composite presented a typical three-stage strain-hardening response and failed due to yarns’ rupture at an average textile exploitation ratio of 0.46. Longo et al. (2020) [4] studied the tensile response of a glass-fiber TRAAM which included expanded glass aggregates in a matrix based on fly ash and metakaolin binders and a sodium-based activator. It was concluded that the developed material had similar mechanical properties to a hydraulic lime-based commercial counterpart while presenting lower thermal conductivity; thus, it has the potential of being applied to structural and energy retrofitting schemes. This was verified by structural and energy analyses of retrofitted masonry elements. Trindade et al. (2017) [5] evaluated the influence of elevated temperatures on the tensile behavior of jute fiber TRAAM with a matrix comprising a metakaolin binder and a sodium-based solution. The researchers found that the type of aggregates incorporated in the mixture—natural quartz sand or chamotte—play a crucial role in the performance of the composite. The results indicated that a strain-hardening tensile response can be obtained by utilizing natural fiber reinforcement, rendering the developed material even more eco-friendly. In addition, the residual compressive strength of the matrix as well as the tensile strength of the corresponding composite, made of refractory aggregates, were superior after exposure to high or elevated temperatures, respectively.

### 1.4. Flexural and Impact Performance of TRAAM

Le Chi et al. investigated the flexural behavior of thin TRAAM plates made of carbon (2018 [6], 2021 [7]) or basalt textile (2019 [8]) embedded in a metakaolin-based matrix reinforced with chopped basalt fibers. The experimental results showed that the mechanical strength of the plates increased with an increase in the number of textile layers and the content of chopped fibers included in the matrix. The flexural performance was characterized by a deflection-hardening response for all or in most specimens reinforced with carbon or basalt textile, respectively. In addition, the failure mode was due to yarns’ slippage from the matrix or yarns’ rupture for most of the specimens with carbon textile or all basalt-furnished ones, respectively. Based on the results, the researchers recommended the use of short, dispersed fibers and meshes with narrow openings. The flexural capacity of two TRAAM systems including short dispersed PVA fibers was studied by Shaikh and Patel (2018) [9], and it was found to be very similar to a cement-based counterpart composite. The two TRAAM systems shared the same AR glass textile and comprised various matrices, namely a fly-ash-based mortar cured at 60 °C for 24 h immediately after casting and a mortar based on a fly ash/slag blend cured at ambient conditions (covered with plastic sheet after casting and left in open air in the lab for 24 h). The results evidenced that the inclusion of PVA fibers improves the energy absorption and leads to sufficient deflection hardening; nevertheless, an increase in PVA fibers while improving the deformation capacity does not have a beneficial effect on the flexural strength. It was also shown that the heat-cured TRAAM system was characterized by higher flexural strength but lower deflection at peak load in comparison to the ambient-cured one. Li et al. (2017) [10] investigated the flexural behavior of hybrid basalt/steel fiber TRAAM panels exposure to high temperatures (400 °C, 600 °C, and 800 °C for durations of one and two hours). The results showed that the panels exhibited deflection hardening and that the flexural performance deteriorated as the exposure temperature and duration increased due to the increasing decomposition and phase transformation of the alkali-activated slag-based mortar, which—in turn—led to the deterioration of bond between basalt fibers and matrix. Finally, the effect of molarity of the sodium hydroxide activator on the flexural capacity of glass-fiber TRAAM based on metakaolin and fly ash was studied by Al Jaberi et al. (2022) [11]. It was found that increasing the molarity of the activator in the AAM binder from 8M to 12M resulted in increased compressive and flexural strength by 20% and 29%, respectively. Consequently, regarding the textile-reinforced specimens the increase in the molarity of matrix led to improved flexural properties in terms of first crack and ultimate loads.

The impact and post-impact behavior of TRAAM composites reinforced with carbon, E-glass, and basalt fabrics were investigated by Samal et al. (2016) [12] implementing out-of-plane low velocity impact test and four point bending tests thereafter. The results showed that E-glass composites had a smaller damaged area compared to carbon composites, while basalt composites had poor impact resistance and low bending strength.

### 1.5. Bond Properties of TRAAM

The bond properties of TRAAM were first studied by Tamburini et al. (2017) [13] who assessed a metakaolin-slag-sodium-silicate grout as a matrix for bonding textiles made of basalt, glass, and carbon fibers or unidirectional steel cords fabrics to clay bricks. The developed matrix was physically, chemically and mechanically characterized, and it was found that it presented good chemical/physical stability, high compressive strength (45 MPa at 28 days), and satisfactory durability against freeze–thaw cycles and high temperatures. Excellent adhesion strength to the soft mud and strong extruded clay bricks was also shown by pull-off tests, irrespective of fiber reinforcements. The shear bond capacity of carbon fiber TRAAM (based on a blend of fly ash/ground granulated blast furnace slag) applied on concrete was tested by Obaida et al. (2021) [14] using the single-lap/single-prism setup. The obtained results were compared with those of a TRM system composed of the same textile and a cementitious matrix with compressive strength identical to that characterizing the alkali-activated matrix. It is noted that various bond lengths were examined for both composite systems (from 50 mm to 300 mm with a step of 50 mm), while the specimens’ repeatability was one or two. The observed failure modes included debonding at the textile-to-matrix interface (with or without matrix cracking) or yarns’ rupture. It was found that the maximum attained bond load corresponding to the TRAAM system was higher than that of the cementitious TRM system for all bond lengths examined, except for that of 250 mm, for which, however, the difference was statistically insignificant. Askouni et al. (2021) [15] examined glass TRAAM using normal-weight and lightweight matrices (with the latter including expanded glass aggregates) based on metakaolin and fly ash. The bond of the TRAAM systems with masonry was compared with that of cementitious counterparts sharing the same textile; matrices (alkali-activated and cement-based ones) had comparable flexural strengths, per density class. The residual bond capacity of these systems was also examined after exposure of TRM-reinforced masonry prisms to 200 °C and 400 °C, for one hour. Failure was always due to sleeve fibers’ rupture along with yarns’ slippage from the mortar, while the lightweight matrices were more prone to cracking. It was concluded that the bond strength of all specimens reduced linearly with increasing temperature, reaching approximately 50% of the reference (non-heated) strength after exposure to 400 °C; for all exposure temperatures considered, residual bond capacities were higher for the TRAAM systems in comparison to that of the cementitious ones. It was also found that the lightweight TRAAM offered better heat protection to the TRAAM-to-masonry interface compared to the normal-weight one. Finally, Skyrianou et al. (2024) [16] employed the modified beam test setup to study the out-of-plane bond of a TRAAM and a conventional TRM system with concrete substrate. The TRAAM system consisted of a binary fiber-reinforced matrix based on metakaolin plus rotary kiln dust and a dry carbon fiber textile. The conventional TRM system was made of the same textile, and its matrix was a cement-based mortar with a flexural strength comparable to that of the alkali-activated mortar. Various bond lengths were examined for both composites, ranging from 50 mm to 150 mm. The failure mode of all specimens was due to fibers’ slippage within the matrix. It was found that the bond strength of TRAAM was higher by 25–94% than that of the conventional TRM, for all bond lengths considered.

The study of Donnini et al. (2024) [17] focused on the comparison of cement- or lime-based (conventional) TRM with alkali-activated ones based on metakaolin or fly ash. All types of matrices were combined with impregnated glass or basalt fiber textile. In addition to the tensile strength and the in-plane bond to clay bricks of the conventional TRM and TRAAM systems, the energy and environmental performance of these systems were also examined. Based on the mechanical characteristics, it was found that the developed TRAAM systems can be a promising alternative to the conventional TRM ones. Furthermore, the estimation of the energy consumption and CO_2_ emissions during the production of the constituent materials of the conventional TRM and TRAAM systems indicated that a fly-ash-based TRAAM can be the most eco-friendly choice, irrespective of the textile used.

### 1.6. Environmental Properties of TRAAM

Various studies have adopted AAM owing to the positive environmental impact (compared to cement-based products), several of which have also presented Life Cycle Assessment (LCA) data that support these claims (e.g., [18,19,20]). However, recent, thorough studies on the environmental impact of various geopolymers showed that the improved effect of AAM is not so straightforward. Kryvenko et al. (2024) [21] and Donnini et al. (2024) [17] highlighted the role of the activator on the overall environmental impact of the mortar, especially when sodium-based activators are adopted, since their production is highly energy costly. The precursors also play a major role, as well, since metakaolin-based AAM may include more embodied energy and carbon than conventional lime-based mortars, whereas fly-ash-based mortars are more energy efficient [17]. Finally, other parameters, such as the methodology used for the LCA (including the database, the software, and the type of allocation per material or process), the source location and the transportation costs of materials, play a major role in the results concerning the environmental impact of the studied material, resulting in huge variations on the carbon footprint of geopolymers [22]. Indicatively, a range of values was provided by Nikravan et al. (2023) [22] regarding the carbon footprint of geopolymer mortars; this was found 8% to 80% lower than that of OPC. The environmental impact of AAMs production is multiparametric and renders their comparison with OPC ones difficult. However, the majority of the currently available data supports that the overall environmental footprint of an AAM matrix is lower than that of a conventional OPC matrix (either slightly or highly) [23,24]; hence, TRAAM could still, generally, be considered as a more sustainable option than conventional (cement-based) TRM.

### 1.7. Scope and Objectives of the Present Study

**Figure 1:**
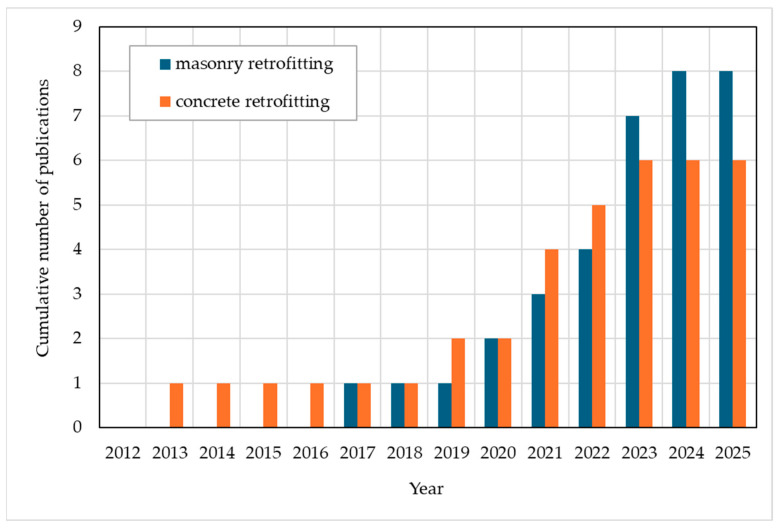
Cumulative number of publications concerning structural retrofitting of masonry and concrete members with TRAAM over the last 13 years.

In summary of these results, it is concluded that TRAAM can develop the favorable strain-hardening response that characterizes the tensile constitutive law of conventional TRM systems provided that an adequate fiber volumetric ratio is used. In addition, a deflection-hardening response can be developed during the flexural performance of TRAAM. Furthermore, the bond strength of TRAAM with various substrates can outperform that of the conventional TRM systems made of matrices with comparable compressive or bending strength. Owing to the promising mechanical behavior of TRAAM and its potential eco-friendly energy and environmental performance, several researchers have studied the application of alkali-activated (fiber reinforced) mortars in various schemes of structural interventions (e.g., Abulencia et al. 2021 [20], Kryvenko et al. 2024 [21]). However, there is a relatively limited number of studies where researchers combined fibrous textiles with AAM matrices for the implementation of TRAAM-retrofitting interventions in concrete and masonry members; these are the studies that the current review article discusses. The focus is directed in the mechanical response the TRAAM-strengthened members and, where relevant data exist, this response is compared with that of members strengthened with conventional TRM systems (lime- or cement-based ones). Most of these studies were published after 2018; therefore, this is a relatively new topic that has drawn increasing attention from researchers during the past few years. The publication timeline of the studies discussed in this review is presented schematically in Figure 1.

The main purpose of this paper is to provide readers with a comprehensive review of the early results of these investigations, in order to acknowledge the various strengths and weaknesses of retrofitting structurally deficient elements with TRAAM instead of conventional TRM. An additional goal of this review is to identify the current knowledge gaps and propose directions for future research on this particular topic. Finally, this review aims to highlight the lack of consensus in the scientific community regarding the testing methods, which is necessary for a more systematic way of performing research on this topic.

## 2. Masonry Members

The current section presents the studies investigating the potential of TRAAM as strengthening means of masonry members. Most of these studies experimentally examined the response of TRAAM-strengthened walls under diagonal compression, in- or out-of-place bending and shear [25,26,27,28]. In one study, some of the strengthened specimens had been exposed to elevated/high temperatures prior to mechanical testing [27]. The combination of TRAAM overlays with thermal insulation EPS/XPS boards for the seismic and energy upgrading of wall specimens or masonry infilled frames was also studied experimentally [29,30,31]. Finally, the possibility of using a lightweight TRAAM system as both a strengthening and thermal insulation overlay of masonry vaults was investigated in a study presenting analytical results and numerical experiments [4]. The presented studies that examine various TRAAM systems for use as external reinforcement of masonry members and, in some cases, also suggest their combination with thermal insulation boards in various sandwich configurations, provide good indications for the prospect of TRAAM successful application in structural and energy upgrading interventions.

### 2.1. Upgrading Response to Diagonal Compression

The current section focuses on studies that examine the response of TRAAM-strengthened wallettes to diagonal compression. In one case, the capacity of these specimens is also compared to that of specimens strengthened with a “conventional” cement-based TRM system. An overview of the discussed studies is presented in Table 1, which includes the type of loading (cyclic or monotonic), the specimens’ characteristics, the constituent TRM materials, the failure mode of the strengthened specimens, and their strength ratio. The latter has been computed as the ratio of the average strength of a group of identical strengthened specimens to that of the control (un-strengthened) ones.

Cholostiakow et al. (2023a) [25] constructed single-leaf wallettes using ridge-faced hollow clay bricks as masonry units and cement/lime-based mortar for the joints. Some wallettes remained un-strengthened to be used as control specimens. The composites adopted for the strengthening of the remaining wallettes consisted of cementitious or alkali-activated matrix and coated basalt- or glass-fiber textile. Both matrices were combined with each textile for the application of one-sided cement-based TRM (TRCM) or TRAAM overlays onto the specimens, while some additional specimens were strengthened with two-sided glass TRAAM overlays. It is noted that the one-sided strengthened specimens were equipped with a double-textile layer overlay, while the two-sided reinforced ones received a single-textile layer overlay (one textile layer on each side). The matrix of the TRAAM systems consisted of metakaolin (precursor) and potassium silicate solution (activator), while it was reinforced with polypropylene fibers. The cementitious and the alkali-activated matrix were characterized by comparable workability and flexural strength. The number of identical specimens per group was equal to three. The control specimens failed due to a splitting step crack along the joints. The unilaterally TRCM-strengthened specimens exhibited failure of the composite overlay (including fiber rupture or/and fiber slippage within the matrix), while those strengthened with TRAAM suffered partial detachment of the overlay from the substrate. It was also observed that among the one-side strengthened specimens, the basalt and glass TRAAM-strengthened ones presented 75% and 55% larger shear strain at peak than their TRCM-strengthened counterparts, respectively. The two-sided TRAAM-strengthened specimens exhibited both failure and partial detachment of the overlay, while their response was more ductile than that of their one-sided strengthened counterparts [specifically, they presented almost double ductility ratio = (strain at peak/strain at cracking)]. Based on their results, the authors concluded that TRAAM could be a promising retrofitting choice for masonry infills of RC frames, since TRAAM-strengthened walls presented similar (in terms of strength, see Table 1) or even better (in terms of deformation capacity) mechanical response than the walls strengthened with TRCM.

Libre et al. (2023) [26] constructed single-leaf wallettes using clay bricks and cement-based mortar. Some wallettes remained un-strengthened to serve as control specimens. Other wallettes were strengthened either on one side or on both, with a TRAAM system comprising one layer of a bamboo-fiber textile. The matrix of the TRAAM system contained a blend of binders, namely mill-scale powder and low calcium Class F fly ash, while sodium hydroxide and sodium silicate (waterglass) were used as activators. It is noted that the alkali-activated matrix was reinforced with chopped bamboo fibers. The natural fibers used for both the reinforcement of the matrix and the construction of the textile had been previously immerged in 10% aluminum sulfate solution to improve the bond between them and the matrix. The rest of the wallettes were covered on one or both sides with the fiber-reinforced alkali-activated mortar used as matrix of the TRAAM system. The number of identical specimens per group was equal to five. The control specimens failed due to cracking along the joints. The one-sided and two-sided TRAAM-strengthened specimens exhibited diagonal cracking, while it was pointed out that some of the former specimens suffered TRAAM-to-substrate detachment. Based on the experimental results, it was concluded that the ductility ratio of the TRAAM-strengthened specimens was enhanced (by 16%) compared to that of the control ones; this phenomenon was more intense in the case where the TRAAM overlay was applied on both sides of the wallettes (i.e., the two-sided strengthened specimens presented 50% higher ductility ratio than the control ones).

Finally, Arce et al. (2024) [27] prepared single-leaf wallettes using solid clay bricks and lime-based mortar. Some un-strengthened wallettes were used as control specimens. The rest of the wallettes were reinforced on both sides with single or double TRAAM overlays that contained one or two dry carbon-fiber textile strips, respectively. The matrix of the TRAAM systems consisted of ferronickel slag and silica fume activated by a solution of potassium silicate and potassium hydroxide. It is also noted that Arce et al. 2024 [20] used ferronickel sand as fine aggregate. The number of identical specimens per group was equal to two. After the application of TRAAM and before mechanical testing a number of specimens were placed in a furnace and were exposed to an air temperature of 300 °C and 550 °C for 60 min (heating rate up to the attainment of the steady-state phase: 50 °C/minute). The remaining TRAAM-strengthened specimens were not exposed to elevated/high temperature prior to testing. The failure mode of the control specimens was due to sliding shear cracks along bed and head joints, suggesting poor bond at brick-to-mortar interface. The failure mode of the strengthened specimens (both unheated and heated) was due to diagonal cracking, top mortar delamination and—for double-layered TRAAM—extensive detachment at the TRAAM-to-substrate interface. It was found that—compared to the control specimens—the strengthened ones presented significantly higher shear strength (see Table 1) and ductility ratios (+373% or +361% for single- or double-layered reinforcement, respectively), as well as higher shear modulus (+23% or +39% for single- or double-layered reinforcement, respectively). Heat exposure had little effect on the residual strength of the heated/strengthened specimens compared to the respective unheated/strengthened ones; on the contrary, the specimens’ stiffness and pseudo-ductility were adversely affected after exposure to 550 °C. Regarding the effect of the reinforcement amount, this was more important than heating for the shear strength of the specimens, while it was almost of the same importance for their shear modulus and pseudo-ductility.

**Figure 2:**
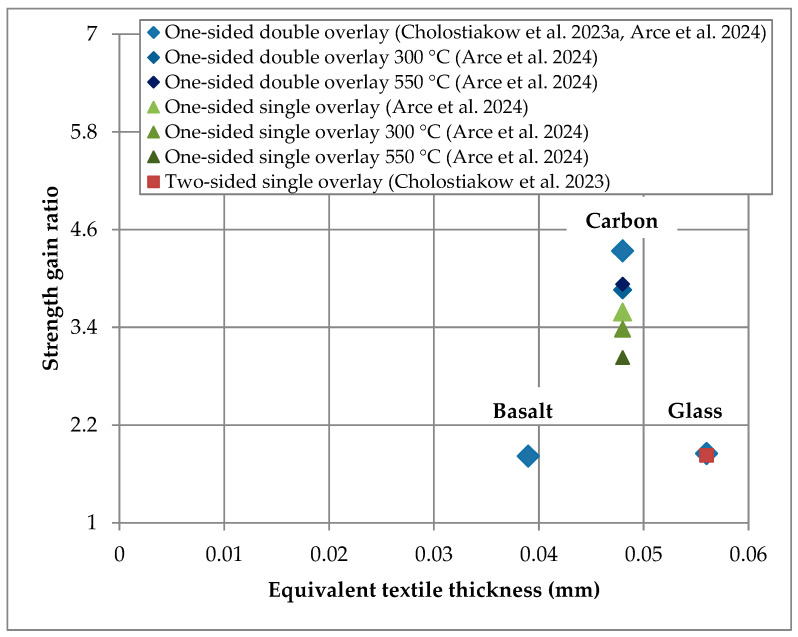
Strength gain ratio versus equivalent textile thickness of TRAAM-strengthened walls subjected to diagonal compression [25,27].

**Figure 3:**
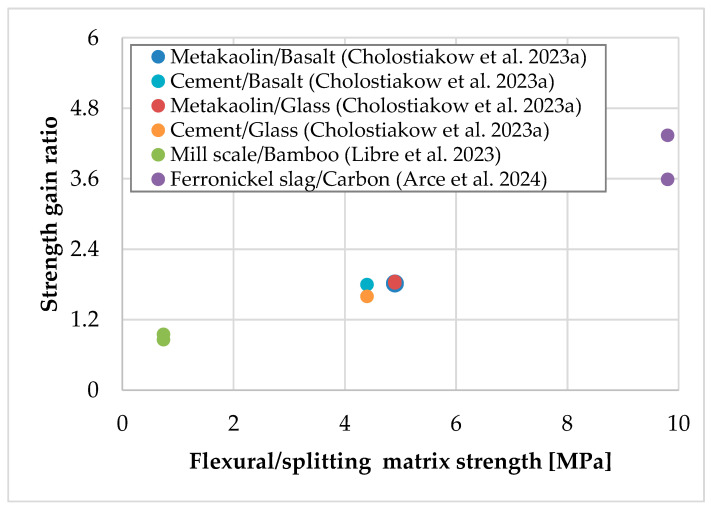
Strength gain ratio versus flexural/splitting matrix strength of TRAAM- and TRCM-strengthened walls subjected to diagonal compression [25,26,27]. (Note: all overlays’ configurations have been included for each TRM system.)

Except for presenting the experimental data in Table 1, the strength gain ratio versus the equivalent textile thickness of the TRAAM-strengthened walls subjected to diagonal compression are also presented in Figure 2 (it is noted that the data of Libre et al. (2023) [26] are absent since the equivalent textile thickness was not provided by the authors). It is observed that the higher strength gain is related to the carbon fiber textile, even in the case of heated strengthened walls. In addition, it appears that the increase (doubling) in the reinforcement amount provided by the carbon TRAAM overlay leads to an increase in the strength gain, while the application of a two-sided instead of a one-sided glass TRAAM overlay with the same reinforcement amount does not affect this parameter. It is also concluded that the strength gain ratio cannot be correlated with the equivalent textile thickness when textiles with different fibers are considered. However, the same does not apply if flexural/splitting strength of the matrices is considered, since irrespective of the mortar’s type, the strength gain ratio increases as the mortar’s strength increases. The correlation of the strength gain ratio with the flexural/splitting matrix strength is given in Figure 3, and it appears to be about the same if the matrix compressive strength is considered (see Table 1).

Finally, based on the conclusion of all related studies, it is highlighted that the ductility ratio of the TRAAM-strengthened walls subjected to diagonal compression is enhanced when compared to that of the un-strengthened (control) walls. Regarding the same parameter and based on the study of Cholostiakow et al. (2023a) [25], it appears that the basalt/glass TRAAM overlays outperform when compared to its TRM counterparts, while the results of Libre et al. (2023) [26] suggest that the two-sided bamboo TRAAM overlay is more effective than the one-sided one. The study of Arce et al. (2024) [27] suggests that the doubling of the reinforcement amount provided by the carbon TRAAM overlay does not substantially affect the enhancement of the ductility ratio.

### 2.2. Upgrading Flexural or Shear Capacity

This section examines the response of TRAAM-strengthened stone walls to in- or out-of-plane bending and shear. In the context of this study, the capacity of the TRAAM-strengthened specimens is also compared to that of specimens strengthened with a “conventional” lime-based TRM system. An overview of the study is presented in Table 2, which includes the type of loading, the specimens’ characteristics, the constituent materials of the adopted TRM systems, the failure mode of the strengthened specimens, as well as their maximum load ratio. Specifically, the maximum load ratio has been computed as the ratio of the maximum load of each strengthened specimen to that of the control (un-strengthened) one.

Gkournelos et al. (2022) [28] studied the in- and out-of-plane response of double-leaf walls subjected to cyclic loading. The authors constructed the walls using natural stone and cement/lime-based mortar, while they adopted two TRM strengthening. The conventional TRM system consisted of lime-based matrix and natural flax-fiber textile (TRLM), while the TRAAM one consisted of an alkali-activated matrix and coated basalt-fiber textile. Regarding the alkali-activated matrix, metakaolin and ladle furnace slag were used as precursors, while potassium silicate solution and potassium hydroxide pellets were used as activators. The experimental program contained in- and out-of-plane bending tests of slender strengthened walls, as well as in-plane shear tests of squat strengthened walls, while un-strengthened counterpart walls (control specimens) were also subjected to the same tests. It is noted that during each test, axial force was applied to the specimens. The examined parameter for all specimens’ configurations was the type of strengthening system. The effect of axial load was additionally examined for the slender specimens subjected to in plane loading, while the thickness of the wall and the positioning of the TRAAM system were also examined for the slender specimens subjected to out-of-plane loading and the slender or squat specimens subjected to in plane loading, respectively. The control specimens subjected to in-plane loading failed due to rocking, while the respective specimens under out-of-plane loading exhibited flexural failure. All TRAAM-strengthened specimens failed due to tensile failure of the textile. The specimens strengthened with the TRLM overlay suffered textile slippage within the matrix. The authors concluded that both strengthening systems resulted in the increase in both strength (see Table 2) and stiffness (the increase being in general larger for TRAAM-strengthened specimens). Regarding the TRAAM system, the premature failure of the specimens was also attributed to the capacity loss of the basalt textile within the alkaline environment of the matrix. The authors suggested that a more appropriate coating of the textiles could provide alkali protection to both types of fibers and could also lead to elimination of the natural fibers’ slippage.

### 2.3. Combination of TRAAM with Thermal Insulation

This section covers the studies that examine the in- or out-of-plane bending response of walls or masonry infills strengthened with integrated systems that contain both TRAAM overlays and thermal insulation boards. The capacity of the TRAAM-strengthened/insulated walls is also compared to that of specimens strengthened with “conventional” cement-based TRM systems. An overview of the studies is presented in Table 3, which includes the type of loading, the specimens’ characteristics, the TRM constituent materials, the failure mode of the strengthened specimens, as well as their maximum attained load.

The combination of TRAAM with thermal insulation boards was employed by Cholostiakow et al. (2023b) [29] for the structural and energy retrofitting of masonry walls representing infills of reinforced concrete (RC) frames subjected to out-of-plane bending. The authors constructed the walls using hollow fired-clay bricks and cement/lime-based mortar. Two matrices were adopted, each combined with two different textiles. The conventional matrix was a cement-based one, while the alkali-activated matrix was fiber-reinforced containing metakaolin as precursor and potassium silicate solution as activator. The cementitious and the alkali-activated matrices were characterized by comparable workability and flexural strength. The textiles used were coated and made of basalt or glass fibers. The experimental program included walls strengthened on one side with a double-layered TRCM or TRAAM overlay. It also included walls in which the strengthening system was combined with EPS boards positioned either between the wall and the TRCM/TRAAM overlay or between the two layers of the TRCM/TRAAM overlay (“sandwich” strengthening configuration). The specimens without insulation or the insulated ones with the “sandwich” configuration exhibited diagonal shear failure. The failure of the specimens in which the insulation was directly bonded to the wall was governed by the detachment at the EPS boards-to-masonry interface. It was concluded that both the TRCM and the TRAAM system were more effective in terms of post-cracking stiffness and ultimate capacity when combined with the EPS insulation, due to the increase in the lever arm. Between the two configurations of the strengthened/insulated specimens the “sandwich” one was the most effective in terms of strength. In addition, it was found that the TRAAM systems presented comparable performance to that of the TRCM ones in the case of the basalt textile, while the glass textile was characterized by poor bond with the alkali-activated matrix (the maximum load of glass TRAAM-strengthened and glass TRAAM-strengthened/insulated specimens with “sandwich” configuration was around 50% lower than that of their TRCM-strengthened counterparts, see Table 3). Furthermore, Cholostiakow et al. (2023c) [30] prepared two nearly half-scale masonry infilled RC frames to study their out-of-plane bending response. The first frame remained un-strengthened serving as the control one. The second frame was strengthened on one side using the basalt TRAAM system adopted by Cholostiakow et al. (2023b) [29] in combination with EPS boards, according to the aforementioned “sandwich” configuration. During testing, each frame was restrained from lateral movements so that only its infill would resist the lateral load that was applied to the center of the infill. The failure of the control RC frame was characterized by a symmetric cracking pattern propagating from the infill’s center towards the frame members. The strengthened/insulated RC frame presented significantly higher (+169%) initial stiffness and almost triple out-of-plane strength (see Table 3) compared to the reference one. The attainment of the latter frame’s maximum load was combined with the detachment of the TRAAM system from the beam, while after failure and load drop by about 18% the outer TRAAM overlay was cracked vertically along the centerline of the infill.

Kapsalis et al. (2025) [31] studied the response of strengthened/insulated walls subjected to out-of-plane monotonic or in-plane cyclic bending. The walls were made of ridge-faced clay bricks and cement/lime-based mortar, while they simulated load-bearing lintels. The authors adopted two composite systems that shared the same dry carbon-fiber textile and were combined with XPS boards. The conventional TRM system consisted of a cementitious matrix that was a commercial product typically used as adhesive for thermal insulation boards (TRCM). The TRAAM system consisted of an alkali-activated matrix identical to that adopted by Arce et al. (2024) [27] (ferronickel slag and silica fume activated by a potassium silicate/potassium hydroxide solution). The combination of the TRCM or TRAAM system with the XPS boards followed two configurations: in the first one, the board was applied directly onto the wall and a double-layered TRAAM overlay was applied externally (M_XPS_TRAAM configuration), whereas in the second, a double-layered TRCM or TRAAM overlay was in contact with the wall and the boards were positioned on top of the overlay (M_TRCM/TRAAM_XPS configuration). Except for the strengthened/insulated walls, two additional un-strengthened walls were constructed so that each could be used as control specimen for each loading case. The failure mode of the control specimen subjected to out-of-plane monotonic bending was due to the formation of a crack at mid-span resulting in the splitting of the wall into two fragments. The corresponding specimens strengthened with the M_TRCM/TRAAM_XPS failed due to a flexural crack that extended almost through the entire depth of the wall along with the debonding at the textile-to-matrix interface. In the case of the M_TRCM/TRAAM_XPS configuration, both strengthening systems increased the out-of-plane bending capacity of the walls, with the TRCM to be more effective than the TRAAM (see Table 3). The failure of the TRAAM specimen strengthened with the M_XPS_TRAAM configuration was due to fibers’ rupture and textile-to-matrix debonding. The M_XPS_TRAAM configuration resulted in the largest increase in the wall’s out-of-plane bending capacity due to the longer level arm of the fibrous reinforcement (see Table 3). The control specimen subjected to in-plane cyclic bending failed due to the development of flexural cracks (propagating through bricks, mortar, and brick-to-mortar interface) near the mid-span. The corresponding specimens strengthened with the M_TRCM/TRAAM_XPS configuration failed due to debonding at the textile-to-matrix interface near the mid-span of the wall. The specimens strengthened with TRAAM suffered fiber rupture regardless of the position of the XPS, followed by limited compressive failure of the wall and the alkali-activated matrix. The increase in the in-plane bending capacity of all strengthened walls was comparable (irrespective of the type of strengthening system and the position of insulation, see Table 3). The deflection at failure of the TRAAM-strengthened specimens with the M_TRAAM_XPS configuration was 64% or 52% lower than that of the corresponding specimens strengthened with the TRCM and subjected to out-of- or in-plane bending, respectively. The M_XPS_TRAAM configuration resulted in the increase in specimens’ deflection at failure by 19 and 17 times compared to the control specimens, for out-of- and in-plane bending, respectively. Finally, the authors noted that the TRAAM system was more prone to fiber rupture than the TRCM ones when bending-induced effects took place, since the alkali-activated matrix had higher stiffness compared to the cementitious one.

**Figure 4:**
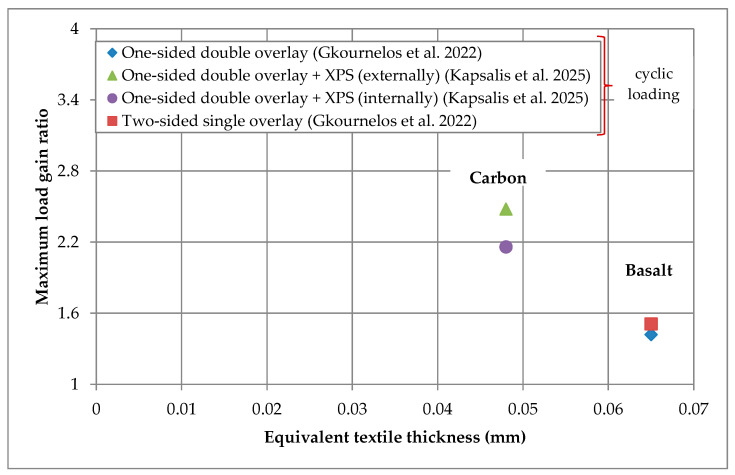
Maximum load gain ratio versus equivalent textile thickness of TRAAM-strengthened walls subjected to in-plane bending (Note: Regarding the study of Gkournelos et al. 2022, data related to BTRAAM1L_M_BTRAAM1L-V0 specimen are not considered.) [28,31].

**Figure 5:**
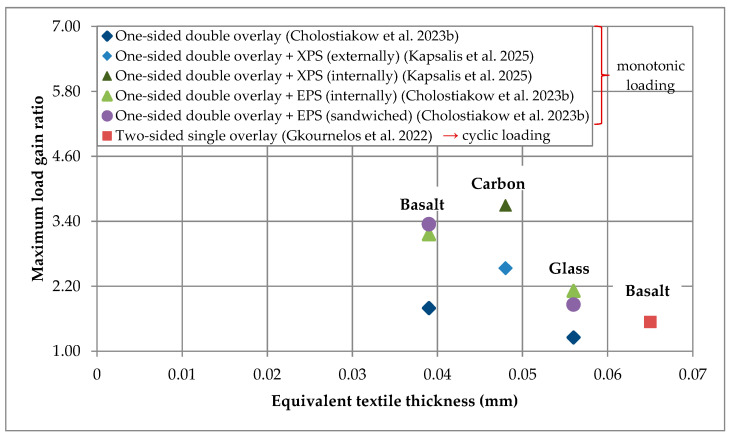
Maximum load gain ratio versus equivalent textile thickness of TRAAM-strengthened walls subjected to out-of-plane bending (Note: Regarding the study of Gkournelos et al. 2022 [28], data related to BTRAAM1L_M_BTRAAM1L-th specimen are not considered.) [28,29,31].

Most of the results presented in the current and previous section are gathered in Figure 4 and Figure 5 that depict the maximum load gain ratio versus the equivalent textile thickness of the TRAAM-strengthened walls subjected to in- and out-of-plane bending, respectively. It is noted that the results of Figure 4 regard specimens subjected to cyclic in-plane loading, while those of Figure 5 represent specimens subjected to monotonic out-of-plane loading, except in the case of wall reinforced with the two-sided basalt TRAAM overlay that was subjected to cyclic loading (i.e., [28]). It is also pointed out that in the absence of a control specimen in the study of Cholostiakow et al. (2023b) [29] (i.e., un-strengthened wall), the related load gain ratios have been computed based on the theoretically estimated value of that specimen’s maximum load. Regarding specimens subjected to in-plane bending (Figure 4), it appears that those strengthened with a carbon TRAAM overlay present higher load gain. It is also observed that the combination of TRAAM overlays with insulation boards can potentially limit the efficiency rate of the strengthening system when boards are applied between the substrate and the overlay (internally). In the case of out-of-plane bending (Figure 5) and regarding the presence and position of insulation boards in relation to TRAAM overlays, it is verified that specimens with no insulation or with boards applied on top of the TRAAM overlays (externally) present lower load gain ratio than those in which the board is applied internally or between two TRAAM overlays. As mentioned above, this fact is attributed to the longer lever arm from the axis of rotation to the TRAAM overlay. In addition, the comment about the relation of the carbon fiber textile to the highest load gain ratio still applies when TRAAM-strengthened walls are subjected to out-of-plane bending. Finally, it is concluded that the strength gain ratio cannot be correlated either with the equivalent textile thickness when textiles with different fibers are considered, or with the flexural/compressive matrix strength (see Figure 4 and Figure 5 and Table 2 and Table 3).

### 2.4. Lightweight TRAAM

Longo et al. (2020) [4] investigated a lightweight TRAAM system as both a strengthening and thermal insulation overlay of masonry vaults through numerical experiments. It is reminded that the adopted TRAAM system consisted of a dry glass-fiber textile and an alkali-activated matrix based on fly ash and metakaolin binders and a sodium silicate plus sodium hydroxide solution. The use of expanded glass beads as fine aggregates rendered the alkali-activated matrix a lightweight mortar (dry mass density 1031 kg/m^3^) with low thermal conductivity (0.222 W/mK). Based on analytical and numerical results, it was concluded that a masonry vault strengthened with the proposed TRAAM system could be safe against the formation of plastic hinges and better thermally insulated compared to a vault filed with a common masonry infill.

### 2.5. General Remarks

The binders used for the alkali-activated mortars are metakaolin, mill-scale powder and slag (ferronickel/ladle furnace), while additives such as fly ash and silica fume have also been adopted. However, it is noted that among the powders used to produce AAM mortars, the metakaolin is characterized by higher CO_2_ emissions and energy consumption (e.g., [23]). To develop more eco-friendly TRAAM systems future studies could focus on alternative precursors such as ferronickel slag, since the studies of Arce et al. (2024) [27] and Kapsalis et al. (2025) [31] provide promising results. Regarding the activators, only potassium- and no sodium-based solutions have been used as activators. The alkali-activated mortars have been combined with treated or untreated textiles made of artificial fibers (i.e., basalt, glass and carbon fibers), while there is a single study adopting natural bamboo fibers for the manufacturing of the textile (see [25]). The use of natural fiber textiles obviously renders TRAAM systems more sustainable; however, the integrity of such fibers in the alkaline environment of the matrix should be further investigated.

TRAAM overlays can increase the strength and ductility of clay brick masonry subjected to diagonal compression. The existent data suggest that the increase in the flexural/splitting strength of the matrix results to more effective—in terms of tensile strength gain—overlays. It also appears that the use of an appropriate precursor can lead to strength enhancement, even after the exposure of the (already strengthened) masonry to elevated/high temperatures prior to mechanical loading. Furthermore, TRAAM jackets can improve the strength and stiffness of stone walls subjected to in- and out-of-plane bending or shear. Finally, based on the results of in- or out-of-plane flexural tests, it appears that the combination of TRAAM strengthening systems with thermal insulation boards can be successful providing to masonry walls/infills both thermal and structural upgrading.

Based on the comparison of the TRAAM strengthening schemes with the corresponding cement- or lime-based TRM ones, it is concluded that the former can present a comparable effectiveness to the latter. However, care should be taken to select properly treated textiles in order to ensure their long-term resistance to the alkaline environment of the matrix. The study of Gkournelos et al. (2022) [28] suggested that coated basalt textile embedded in a mortar with metakaolin and ladle furnace slag binders as well as with potassium-based solution lost its capacity due to the high alkalinity of the matrix. In addition, Cholostiakow et al. (2023b) [29] noticed poor bond between coated glass textile and mortar with metakaolin-binder and potassium-based solution. Finally, Kapsalis et al. (2025) [31] remark that TRAAM system could be more prone to fiber rupture than the TRCM ones, when the alkali-activated matrix is stiffer than the cementitious one and bending-induced effects take place.

Apart from the experimental results, the studies of Cholostiakow et al. (2023a) [25] and Libre et al. (2023) [26] conducted analytical work to reproduce the experimental results. Regarding the former study, it was concluded that the design provisions adopted (ACI 549.4R-13) overestimate both the load corresponding to the diagonal cracking and the ultimate peak strength of the TRAAM/TRCM-strengthened wallettes subjected to diagonal compression. On the contrary, in the latter study, it was concluded that the provisions of ACI 549.4R-13 could well predict the shear capacity of the TRAAM-strengthened wallettes.

## 3. Concrete Members

Among the studies addressing the rehabilitation/strengthening of structural elements by use of textile-reinforced alkali-activated materials, the following have applied the system to reinforced concrete (RC) members [32,33,34,35,36] or to plain concrete cylinders [37]. Excluding [33,36] (structural upgrading against shear), strengthening in the aforementioned studies aimed to increase either the flexural [32,34,35] or the compressive [37] capacity of the elements rendering the fibrous reinforcement along the direction normal to the principal tensile strains rather idle/ineffective, since it only provides some anchorage for the fiber yarns parallel to the principal tensile strains. Nevertheless, these publications provide good indications for the prospect of successful application of such systems in structural upgrading interventions.

### 3.1. Upgrading Flexural Capacity

As conventional cement-based TRM (namely, TRCM), TRAAM can also be applied to the tension face of RC members such as beams or one-way slabs to enhance their flexural capacity. For both types of elements, the application method is the same and the amount of textile reinforcement has a direct influence on the flexural performance parameters (cracking load, elastic stiffness, yielding and maximum load) in the same manner as TRCM.

Shen et al. (2021) [34] performed four-point bending, static, monotonic tests to mid-scale RC beams strengthened with carbon fiber-based TRAAM. As reported by the researchers, generally, TRAAM strengthening increases the cracking, yielding, and ultimate loads of RC beams. However, despite the addition of short, dispersed polypropylene fibers in the matrix and the relatively high amount of textile reinforcement used in this study (equivalent thickness of applied textiles varied from 0.27 mm to 0.53 mm) the overall gain in flexural strength was rather low varying between 13% to 26%. The results in strength gain, stiffness and cracking load were very similar when a TRCM system was used instead of the TRAAM. Due to the load-controlled way of executing the bending tests, the post-peak branches of the load-deflection curves were not available; hence, there is no information on the gains in terms of deformation capacity. Nevertheless, it was observed that the replacement of the cementitious matrix by the alkali-activated one resulted in shifting the failure mode from debonding at the ends of the TRCM overlay to a mixed failure mode governed by fiber slippage and rupture. These results are contradictory to those of Menna et al. (2013) [32] who tested full-scale RC beams in static, monotonic load by four-point bending, and reported a negligible gain in the load-bearing capacity. The tests of Menna et al. [32] also showed that there is no change in failure mode between the un-strengthened and TRAAM-strengthened beams. According to the authors of this review, the difference in the effectiveness of the two systems does not lie in the significantly lower fiber volume fraction used by Menna et al. [32] (compared to the one used in the tests of Shen et al. [34]), but in the very low utilization of the carbon fibers in the tests of Menna et al. [32]. The researchers of the latter study reported failure by early debonding at the textile-to-matrix interface, meaning that the exploitation ratio of the reinforcement was extremely low. A potential reason behind the differences among the two studies is the different alkali-activated matrix (metakaolin-based precursor with sodium-based activator in the case of Menna et al. [32]; granulated blast furnace slag with fly ash, activated by a sodium-based activator in the case of Shen et al. [34]). It is possible that the metakaolin-based mortar activated by a sodium-based solution did not result in a suitable matrix for the impregnation of carbon fibers (hence, the poor bond between the two materials) despite its very high compressive strength (98 MPa at 28 days). It is noteworthy that in all the other studies that are discussed in this review, where the researchers used a metakaolin-based precursor, activation was always performed by potassium-based solutions. However, further research is required to investigate the effect of the activators on the bond properties of metakaolin-based matrices and carbon fiber textiles, because the current results are very limited to reach solid conclusions.

Zhang et al. (2022) [35] performed four-point bending, static, monotonic tests to full-scale one-way RC slabs strengthened with carbon fiber-based TRAAM. According to the general observations, TRAAM strengthening effectively enhances the flexural capacity and post-cracking stiffness of reinforced concrete slabs. The equivalent thickness of the reinforcing textiles varied between 0.044 mm and 0.133 mm, and the fractional strength gain varied between 26% and 92% depending on the number of textile layers. The respective gains observed when using an equivalent TRCM were approximately 15–20% lower, indicating a better exploitation of the fibers in the case of TRAAM strengthening schemes. Despite the substantial gains in strength, deflections at failure were lower for the strengthened specimens (by 13% to 32% for both systems, i.e., cement-based and AAM-based). Both systems (TRCM and TRAAM) offered a similar enhancement of crack control compared to the non-strengthened specimen. However, strengthening with TRAAM instead of TRCM shifted the failure mode from concrete crushing to slippage and rupture of fibers with smaller and more dense cracks in the tension zone. It is noteworthy that the gain in load-bearing capacity reported in the study of Zhang et al. [35] is significantly higher compared to this in the studies of Shen et al. [34] and Menna et al. [32]; however, this may be related to the effect of geometry. The slabs tested in the study of Zhang et al. [35] were significantly thinner than the beams discussed in the other two studies, meaning that the effect of similar TRAAM jackets can be more pronounced at the slabs. Therefore, it is not possible to reach any solid conclusions regarding the effect of the different fiber volume fractions or the different precursors/activators used in these studies.

### 3.2. Upgrading Shear Capacity

In shear strengthening applications, TRAAM can also be used in the same manner as TRCM; however, this has been verified experimentally by only one study, so far. Zhang et al. (2019) [33] applied a TRAAM system to the shear span of mid-scale RC beams using U-shaped jackets (i.e., three-sided wrapping) to enhance their shear capacity. The tests were performed on a four-point bending setup in a static, monotonic way. The researchers tested the effectiveness of jackets utilizing unidirectional or bidirectional carbon fiber textiles with various yarn spacings, in single or multiple layers. TRAAM strengthening was found to effectively enhance the shear capacity of the beams with strength gain percentages varying between 35% to 127%, depending on the amount of reinforcement, textile architecture and the presence of additional anchoring measures. Regarding the effects of textile architecture, it was found that the contribution of unidirectional fiber strands to shear capacity is less significant compared to that of bidirectional textiles, while a denser mesh pattern in TRAAM strengthening is beneficial for improving the shear capacity without the need for special anchorage measures. Nevertheless, anchoring the jackets with steel strips resulted in further strength gains, which were approximately 15–20% higher than those of equivalent systems without anchorage. Comparing these results to the ones of specimens with equivalent TRCM systems it was found that the effective strain developed in the textile of TRAAM-strengthened beams is comparable to or even exceeds that in TRCM-strengthened beams with similar wrapping configurations. Various failure modes were observed, including fiber rupture at shear cracks and debonding of the strengthening jacket accompanied by delamination of the concrete cover, the latter being observed at schemes with a large amount of reinforcement (three layers, in this study). In addition, delamination was also observed in an FRP-based equivalent system tested by the same researchers, although the failure of the FRP-based system was more brittle than that of the TRAAM-based one. Similar results regarding the failure modes and the effect of the textile amount and architecture are observed for typical TRCM-based shear strengthening schemes [38].

### 3.3. Upgrading Compressive Capacity

Concrete elements can be wrapped with TRAAM layers to provide passive confinement, increasing their compressive strength and deformation capacity in the same manner as with TRCM systems. The application method typically involves wet lay-up on properly prepared surfaces, overlapping the last layer in the hoop direction, as reported in the study of Wang et al. (2021) [37]. The researchers applied TRAAM jackets (based on basalt fiber reinforcement) on plain concrete cylinders of typical dimensions of 150 mm × 300 mm. The cylinders were then tested in compression in a monotonic and cyclic manner. The equivalent thickness of the reinforcing textiles varied between 0.048 mm and 0.144 mm and the fractional strength gains varied between 15% and 70%, depending mainly on the amount of reinforcement. The effect of the loading path (monotonic or cyclic) had a limited influence on the strength gain (varying between 4% and 16%, strength gain always being higher for cyclic tests). The strength of the TRAAM matrix (25 MPa or 55 MPa) also had limited influence on the confined strength gain (varying between 6% and 13%); however, it had a more profound effect on the ductility and energy dissipation of the confined elements. TRAAM jacketing substantially improved the axial deformation, ductility, and energy dissipation ability of the specimens; strain increases at failure varied between 54% and 249% for the confined specimens. Nevertheless, no strain hardening was observed for any tested configuration. The observed failure modes included a wide vertical crack at the textile overlap area and textile-to-matrix debonding with negligible damage to the concrete core when only one textile layer was applied. For more heavily strengthened schemes (two or three textile layers) the cracks were multiple and oblique, while the concrete core was heavily damaged after failure of the specimen. Finally, the researchers compared their results with results of TRCM confined specimens found in the literature and concluded that the efficiency of TRAAM jackets is higher than that of TRCM jackets; this can be attributed to the stronger textile-to-matrix bond of the developed TRAAM in [37]. Nevertheless, more experimental results are necessary before reaching general conclusions.

### 3.4. Seismic Retrofitting

TRM jacketing is an effective way of improving the seismic response of RC elements. Azdejkovic and Triantafillou (2023) [36] applied TRCM and TRAAM jackets to enhance the seismic performance of short concrete columns which, usually, are highly susceptible to earthquake loads and tend to fail in a brittle way due to shear. Tests were conducted on mid-scale specimens by applying a lateral load (simultaneously with an axial load) in a cyclic manner. The reinforcing textiles consisted of carbon fibers, and they were applied in equivalent thicknesses of 0.186 mm and 0.372 mm (two and four layers, respectively). Both systems (TRCM and TRAAM) led to strength gain percentages of approximately 15% and 20% for two and four layers, respectively. A much larger increase in deformation capacity was obtained by both systems, with deformation gains being in the order of 160% and 500%. However, despite the ductility increase neither system prevented the abrupt shear failure, regardless of the number of textile layers (two or four) applied. According to the researchers, the TRAAM-strengthened specimens performed very similarly to the TRCM-strengthened ones, indicating that TRAAM has the potential to be a sustainable alternative to conventional cement-based TRM for seismic retrofitting. Nevertheless, according to the same researchers and based on the experience obtained so far, the applicability of TRAAM is hindered by the limited workability of the mortar, which needs to be improved for in-situ applications. Finally, more experimental results are necessary to validate these conclusions.

### 3.5. General Remarks

Strengthening of RC elements with TRAAM has been investigated only by the adoption of carbon fibers. Basalt fibers have been used only in the study that investigated the confinement of plain concrete cylinders [37]. The use of glass-fiber textile or natural fiber textile-reinforced AAM has not been investigated for strengthening concrete elements. As for masonry retrofitting, metakaolin has been used as precursor in combination with fly ash or silica filler in three of the studies discussed in Section 3. Furnace slags have been used in two studies, while only one study has used a ternary blend consisting of metakaolin, furnace slag and fly ash. Potassium-based activators seem to be preferred for mixtures with metakaolin while sodium-based activators are mostly used for mixtures that adopt furnace slags.

**Figure 6:**
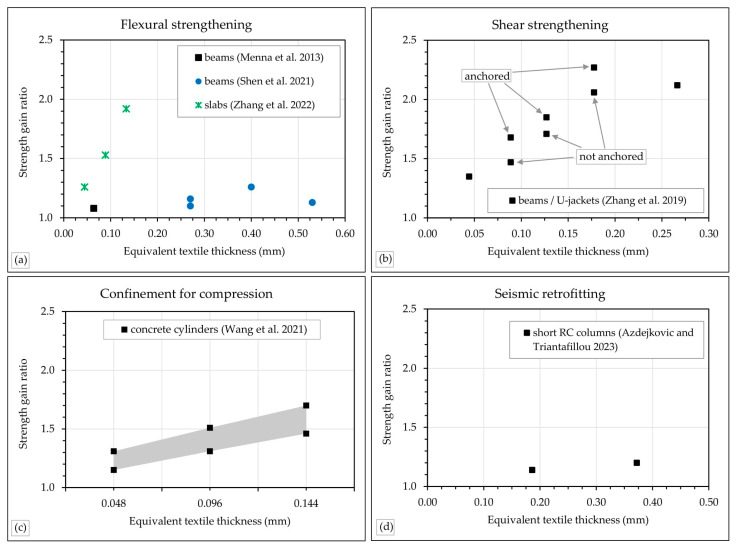
Load gain ratio versus equivalent textile thickness of TRAAM-strengthened concrete elements: (**a**) flexural strengthening; (**b**) shear strengthening; (**c**) confinement for compression; (**d**) seismic retrofitting [32,33,34,35,36,37].

For a visual representation of the effectiveness of the TRAAM systems, the ratios of strength gain are plotted in Figure 6 versus the equivalent thickness of the used textiles. It is observed that, according to the current studies, the effectiveness of carbon TRAAM for flexural strengthening schemes is higher for strengthening slabs than beams, since it can lead to increase in load-bearing capacity of the slab up to 92%, whereas the maximum load gain in beams is equal to 26% and was achieved by a higher amount of reinforcement (compared to the 92% increase in the slab strengthening). Shear strengthening can lead to strength gains that depend mainly on the amount of reinforcement, whereas the presence of anchorage has a noticeable effect, too. Confinement of plain concrete can also be performed effectively with TRAAM with strength gains varying between 15% and 70%, depending mainly on the amount of reinforcement. However, various other parameters, such as the strength of the TRAAM matrix and the loading path also affect the results. A more detailed analysis can be found in [37]. Finally, the results from seismic retrofitting schemes show relatively low strength gain ratios (14% to 20%), although the available data are very limited. However, the effectiveness of the TRAAM systems cannot only be characterized by the strength gain but also from other parameters, too, such as deformation gains and shifting of failure modes into more favorable ones. The effect of these parameters is aggregated in Table 4.

Another interesting observation based on this overview is that the preferred type of fiber for the production of TRAAM is carbon fibers, because they are not susceptible to the high alkalinity of the alkali-activated matrices. The matrices that have been used in the relevant studies were of various compositions (regarding precursors and activators) resulting in various strength grades. This indicates the large potential not only to tailor compositions in terms of constituent materials but also to fine tune mix proportions based on local availability of waste materials or industrial by-products with a direct effect on minimizing production cost. Finally, based on the results of these studies (as discussed in the previous sections) it becomes evident that for all cases of retrofitting concrete members opting for a TRAAM system instead of a conventional TRCM leads to comparable results in terms of strength, deformation and seismic response, in general.

Apart from the experimental results, all the above studies included analytical work in an effort to reproduce the experimental results. The studies of Menna et al. (2013) [32] and Zhang et al. (2022) [35] that investigated the flexural capacity of TRAAM-strengthened elements concluded that existing models for the calculation of load-bearing capacity of FRP-strengthened slabs can be used for TRAAM-strengthened slabs, too, with reliable results, by applying relevant modification factors to the effective strains in fibers accounting for different failure modes. Likewise, Shen et al. (2021) [34] concluded that an efficiency factor for the utilization of the fibers needs to be determined to predict the yielding and failure loads of the TRAAM-strengthened beams with a simple cross-section analysis, while assuming a perfect bond between all materials (as per Euler-Bernoulli’s theory). Similar results were yielded by Zhang et al. (2019) [33] regarding the shear capacity of TRAAM-strengthened beams, who used the model of Escrig et al. (2015) [38] (established for TRM shear strengthening schemes) by calibrating the values of the effective strain of the textiles used in TRAAM. Concerning confinement and seismic retrofitting, the studies of Wang et al. (2021) [37] and Azdejkovic and Triantafillou (2023) [36] concluded that existing models for concrete confined with composite materials yield satisfactory results for TRAAM, as for TRCM. In conclusion, it is evident that there is no need for the development of new analytical methods for the simulation and prediction of the structural capacity of TRAAM-strengthened RC members, since existing models can be used with updated efficiency factors for the fibrous reinforcement; however, additional experimental results are necessary for the calibration and validation of the updated models.

## 4. Conclusions

This paper discusses the current state-of-the-art regarding structural retrofitting of existing masonry and concrete elements with textile-reinforced alkali-activated mortars (TRAAM). The main scope of this review is to highlight the applicability of TRAAM for such interventions and to compare its performance with respect to conventional systems of textile-reinforced mortars—TRM (i.e., non-metallic fibrous reinforcement in the form of open-weave textiles, impregnated in cement- or lime-based mortars). In addition, this review aims to identify the various knowledge gaps and, thus, facilitate further organization and development of experimental and analytical studies that would shed light into the promising field of sustainable retrofitting of concrete and masonry structures.

Based on the overview of the current studies, it is concluded that TRAAM demonstrates a promising potential as a sustainable and effective alternative to cement- or lime-based TRM for retrofitting masonry and concrete. Specifically, regarding masonry retrofitting, based on the findings of this review, the tensile strength of TRAAM-strengthened masonry panels appears to be improved more when alkali-activated mortars with higher strength are adopted as matrices. In addition, regarding the tensile or bending strength of TRAAM-strengthened masonry members, it appears that the use of textiles with carbon fiber results in higher strength gain ratios compared to glass or basalt fiber textiles. The studies that investigated retrofitting of concrete elements indicate that TRAAM exhibits comparable performance in terms of flexural strengthening, shear strengthening, confinement, and seismic retrofitting, with strength gain ratios ranging between 1.1 and 2.4, depending on the application. The early findings indicate that the application of TRAAM retrofitting systems is more effective in flexural strengthening of slabs and shear strengthening of beams. In addition, all studies indicate that existing models can be used for the structural design of retrofitting schemes with TRAAM jackets as long as there is proper calibration of these models with additional experimental data.

The bond strength of TRAAM, either inherent (textile-to-matrix bond) or with the substrate, has been reported to be equivalent or superior to conventional TRM (especially at or after exposure to increased temperatures). This applies to masonry and concrete substrates, although, for concrete substrates this has been derived mainly by visual observations. Therefore, it has yet to be verified and quantified by bond tests on concrete substrates whereas additional bond tests on masonry substrates are also necessary since the currently available studies are very limited. It was also found that the use of TRAAM offers enhanced material properties and better performance at high temperatures; however, this has only been tested on masonry substrates.

Finally, it was concluded that the adaptability of TRAAM in various retrofitting scenarios (reinforcement with natural fibers, combination with thermal insulation, production of lightweight matrices for retrofitting), combined with its comparable or enhanced effectiveness, highlights its potential to revolutionize sustainable construction practices. However, the application of TRAAM can be more complex compared to cement- or lime-based TRM, requiring careful consideration of mixing procedures, curing conditions, and workability. Further research on optimizing the application process (including post-application quality control checks) and developing user-friendly guidelines will facilitate wider adoption of TRAAM. Furthermore, for the development of TRAAM systems with lighter environmental impact, the adoption of alkali-activated matrices based on precursors other than metakaolin and the use of natural fiber textiles are proposed.

In addition to the knowledge gaps mentioned above, research must also cover some ground on the long-term performance of TRAAM composites, including durability, creep, and shrinkage. Further investigations are needed to assess their behavior under various environmental conditions and loading scenarios. Nevertheless, the lack of systematic methods of performing the relevant research by the scientific community should be highlighted and tackled in an organized way (e.g., organization of relevant scientific or technical committees leading to relevant guidelines). Finally, currently, there is a lack of comprehensive standards and design guidelines for TRM retrofitting [39], which becomes even more evident for TRAAM applications. Developing specific standards and guidelines will help engineers and practitioners properly design and implement TRAAM in construction projects. Addressing these knowledge gaps through further, more systematic, research and development will help establish TRAAM as a reliable and sustainable alternative to cement- or lime-based TRM, promoting its wider application in strengthening and retrofitting masonry or concrete structures.

## Figures and Tables

**Table 1:** List of tests regarding the response to diagonal compression of TRAAM/TRCM-strengthened masonry members (all dimensions are in mm; all strength values are in MPa).

Study	Loading	Specimen (Dimensions) {Number}	Matrix Precursor/Activator/Chopped Fibers ^1^ (Flexural or Splitting Strength|Compressive Strength—MPa)	Textile Treatment/Fibers (*t_f_* ^2^—mm)	Group of Specimens ^3^	Failure Mode	Strength Ratio ^4^
Cholostiakow et al. 2023a [25]	Monotonic	Single-leaf wallettes (700 × 700 × 65) {18}	Metakaolin/potassium silicate/P: (4.9_f_|46.0) ^5^ Cement/water/micro PP: (4.4_f_|24.4)	Coated/basalt or glass (basalt: 0.039; glass: 0.056)	M_BTRAAM2L	Partial TRAAM-to-substrate detachment	1.82
					M_GTRAAM2L		1.85
					GTRAAM1L_M_GTRAAM1L	Partial TRAAM-to-substrate detachment + TRAAM failure of TRAAM (fiber rupture or/and fiber slippage within the matrix)	1.83
					M_BTRCM2L	TRCM failure	1.80
					M_GTRCM2L		1.60
Libre et al. 2023 [26]	Monotonic	Single-leaf wallettes (350 × 350 × 50) {25}	Mill-scale powder and fly ash/sodium hydroxide and sodium silicate/bamboo (0.74_s_|3.08)	Immersion in aluminum sulfate solution/bamboo (-)	M_BTRAAM1L	Diagonal cracking (TRAAM failure) or TRAAM-to-substrate detachment (intact TRAAM)	0.95
					BTRAAM1L_M_BTRAAM1L	Diagonal cracking (TRAAM failure)	0.86
Arce et al. 2024 [27]	Monotonic	Single-leaf wallettes (700 × 700 × 10) {14}	Ferronickel slag and silica fume/potassium silicate and potassium hydroxide (9.8_f_|77.9)	Dry/carbon (0.048)	M_CTRAAM1L_20 ^6^	Diagonal cracking, top mortar delamination and—for double TRAAM—extensive detachment at TRAAM-to-substrate interface	3.59
					M_CTRAAM2L_20		4.34
					M_CTRAAM1L_300		3.38
					M_CTRAAM2L_300		3.86
					M_CTRAAM1L_550		3.03
					M_CTRAAM2L_550		3.93

^1^ if any. ^2^ *t_f_* = equivalent thickness of the textile (ratio of the dry-fiber textile weight in each direction to the density of the fibers’ material). ^3^ Notation: M_XYnL or XYnL_M_XYnL corresponds to one-sided or two-sided strengthening, respectively; M stands for the masonry substrate, X is the first letter of textile’s fibers, Y is the type of strengthening system (TRAAM or TRCM), and n equals to the number of textile layers (L). ^4^ Capacity of strengthened specimens over that of the control (un-strengthened) ones. ^5^ Subscript *f*/*s* stands for flexural or splitting strength. ^6^ Exposure to high temperature prior to mechanical testing.

**Table 2:** List of tests regarding the response to bending or shear of TRAAM/TRLM-strengthened masonry members (all dimensions are in mm; all strength values are in MPa).

Study	Test (Loading)	Specimen (Dimensions) {Number}	Matrix Precursor/Activator (Flexural Strength|Compressive Strength—MPa)	Textile Treatment/Fibers (*t_f_* ^1^—mm)	Group of Specimens ^2^	Failure Mode	Maxi Mum Load Ratio ^3^
Gkournelos et al. 2022 [28]	In-plane bending (cyclic)	Slender double-leaf wall (1200 × 400 × 150) {5}	Metakaolin and ladle furnace slag/potassium silicate and potassium hydroxide (5.8|49.2) Lime/water (1.5|4.2)	Coated/basalt or dry/flax basalt: 0.065 flax: 0.229	BTRAAM1L_M_BTRAAM1L	Textile rupture	1.51
					M_BTRAAM2L	Textile rupture	1.42
					FTRLM3L_M_FTRLM3L	Fiber slippage	1.55
					BTRAAM1L_M_BTRAAM1L-V0 ^4^	Textile rupture	0.92
	Out-of-plane bending (cyclic)	Slender double-leaf wall (1200 × 400 × 150/22) {5}			BTRAAM1L_M_BTRAAM1L	Textile rupture	1.54
					BTRAAM1L_M_BTRAAM1L-th ^5^	Textile rupture	2.50
					FTRLM3L_M_FTRLM3L	Fiber slippage	1.48
					FTRLM3L_M_FTRLM3L-th ^5^	Textile rupture	3.18
	In-plane shear (cyclic)	Squat double-leaf wall (1000 × 1100 × 150) {4}			BTRAAM1L_M_BTRAAM1L	Textile rupture	1.62
					M_BTRAAM2L	Textile rupture	1.56
					FTRLM3L_M_FTRLM3L	Fiber slippage	1.19

^1^ *t_f_* = equivalent thickness of the textile (ratio of the dry-fiber textile weight in each direction to the density of the fibers’ material). ^2^ Notation: M_XYnL or XYnL_M_XYnL corresponds to one-sided or two-sided strengthening, respectively; M stands for the masonry substrate, X is the first letter of textile’s fibers, Y is the type of strengthening system (TRAAM or TRLM), and n equals to the number of textile layers (L). ^3^ Capacity of strengthened specimens over that of the control (un-strengthened) ones. ^4^ Specimen without axial load. ^5^ Thicker specimen.

**Table 3:** List of tests regarding the response to in- or out-of-plane bending of TRAAM/TRCM-strengthened/insulated masonry members (all dimensions are in mm; all strength values are in MPa).

Study	Test (Loading)	Specimen (Dimensions) {Number}	Matrix Precursor/Activator/Chopped Fibers ^1^ (Flexural Strength|Compressive Strength—MPa)	Textile Treatment/Fibers (*t_f_* ^2^—mm)	Group of Specimens ^3^	Failure Mode	Maximum Load [kN]
Cholostiakow et al. 2023b [29]	out-of-plane bending (monotonic)	Wall (1090 × 390 × 65) {12}	Metakaolin/potassium silicate/PP (5.5|44.3) Cement/water/micro PP (4.9|22.2)	Coated/basalt or glass (basalt: 0.039; glass: 0.056)	M_GTRCM2L	Shear	7.62
					M_BTRCM2L	Shear	5.97
					M_GTRAAM2L	Fiber slippage/rupture	4.04
					M_BTRAAM2L	Shear	5.79
					M_EPS_GTRCM2L	EPS board debonding	5.25
					M_EPS_BTRCM2L	EPS board debonding	8.94
					M_EPS_GTRAAM2L	Fiber slippage/rupture	6.82
					M_EPS_BTRAAM2L	EPS board debonding	10.2
					M_GTRCM1L_EPS_GTRCM1L	Shear	11.76
					M_BTRCM1L_EPS_BTRCM1L	Shear	9.52
					M_GTRAAM1L_EPS_GTRAAM1L	Fiber slippage/rupture	6
					M_BTRAAM1L_EPS_BTRAAM1L	Shear	10.8
Cholostiakow et al. 2023c [30]	out-of-plane bending (monotonic)	infill of RC frame (1570 × 250 × 64 ) {2}	Metakaolin/potassium silicate/PP (5.8|42.7)	Coated/basalt (0.039)	CONTROL	Infill’s symmetric cracking	16.3
					M_BTRAAM1L_EPS_BTRAAM1L	Detachment of TRAAM from beam	46
Kapsalis et al. 2025 [31]	out-of-plane bending (monotonic)	Wall (1300 × 400 × 85) {8}	Ferronickel slag and silica fume/potassium silicate and potassium hydroxide (7.7|79.7) Cement/water (3.2|9.6)	Dry/carbon (0.048 mm)	CONTROL	Wall splitting	4.79
					M_CTRCM2L_XPS	Wall’s flexural craking + textile-to-matrix detachment	16.36
					M_CTRAAM2L_XPS		12.14
					M_XPS_CTRAAM2L	Fibers’ rupture + textile-to-matrix detachment	17.68
	in-plane bending (cyclic)				CONTROL	Flexural cracks near mid-span	11.15
					M_CTRCM2L_XPS	Textile-to-matrix detachment near mid-span	25.02
					M_CTRAAM2L_XPS	Textile-to-matrix detachment near mid-span + fibers’ rupture	27.6
					M_XPS_CTRAAM2L	Fibers’ rupture	24.1

^1^ if any. ^2^ *t_f_* = equivalent thickness of the textile (ratio of the dry-fiber textile weight in the warp direction to the density of the fibers’ material). ^3^ Notation: M_XYnL or M_I_XYnL or M_XYnL_I_XYnL correspond to various one-sided strengthening/insulation configurations; M stands for the masonry substrate, I represents the insulation (where present), X is the first letter of textile’s fibers, Y is the type of strengthening system (TRAAM or TRCM), and n equals to the number of textile layers (L).

**Table 4:** List of tests regarding TRAAM-strengthened concrete members (in chronological order; all dimensions are in mm; all strength values are in MPa).

Study	Test (Loading)	Specimens {Number}	Matrix Precursor/Activator/Chopped Fibers ^1^ (Flexural Strength|Compressive Strength—MPa)	Textile Treatment/Fibers (*t_f_* ^2^—mm)	Failure Mode	Strength Ratio	Deformation Ratio
Menna et al. 2013 [32]	Flexure (4-point bending/static)	Full-scale beams {5}	Metakaolin/sodium hydroxide and sodium silicate (not provided|98)	Dry/Carbon (0.064)	Concrete crushing; textile-to-matrix debonding	1.08	0.97
Zhang et al. 2019 [33]	Shear (4-point bending/static)	Mid-scale beams {10}	Metakaolin and fly ash/potassium silicate (7.5|43.3)	Dry/Carbon (0.044–0.266)	TRAAM-to-substrate debonding; concrete cover delamination; fiber rupture at shear cracks	1.35–2.27	
Shen et al. 2021 [34]	Flexure (4-point bending/static)	Mid-scale beams {7}	Blast furnace slag and fly ash/sodium hydroxide and sodium silicate/PP (not provided|53.5)	Not provided/Carbon (0.27–0.53)	Slippage and rupture of fibers	1.10–1.26	
Wang et al. 2021 [37]	Compression (monotonic and cyclic)	Cylinders 150 × 300 {28}	Blast furnace slag and fly ash/sodium hydroxide and sodium silicate/PVA (not provided|25–55)	Epoxy resin impregnated/Basalt (0.048–0.144)	Textile-to-matrix debonding; fiber rupture; crushing of concrete core	1.15–1.70	1.54–3.49
Zhang et al. 2022 [35]	Flexure (4-point bending/static)	Full-scale one-way slabs {8}	Metakaolin and fly ash/potassium silicate (7.0|45.1)	Dry/Carbon (0.044–0.133)	Fiber slippage and rupture	1.26–1.92	0.74–>0.87
Azdejkovic and Triantafillou 2023 [36]	Shear (lateral/cyclic)	Mid-scale short columns {7}	Metakaolin, ladle furnace slag and fly ash/potassium silicate and potassium hydroxide/PVA (6.8|40.7)	Dry/Carbon (0.186–0.372)	Fiber rupture in abrupt shear failures	1.14–1.20	2.58–5.99

^1^ if any. ^2^ *t_f_* = equivalent thickness of the textile (ratio of the dry-fiber textile weight in the warp direction to the density of the fibers’ material).
